# Roles of DRP1 and the fission protein interactome as regulators of cellular stability and sarcopenia in skeletal muscle aging

**DOI:** 10.4103/agingadv.agingadv-d-25-00013

**Published:** 2025-12-18

**Authors:** C. Nivedya, Prasanna Venkhatesh, Benjamin I. Rodriguez, Han Le, Jeremiah Afolabi, Andrea Marshall, Kit Neikirk, Sepiso K. Masenga, Muhammad Aftab, Leo Jake Kazma, Prasanna Katti, Antentor Hinton

**Affiliations:** 1 Department of Biology, Indian Institute of Science Education and Research (IISER) Tirupati, AP, India;; 2 Department of Molecular Physiology & Biophysics, Vanderbilt University, Nashville, TN, USA;; 3 Department of Biomedical Sciences, Meharry Medical College, Nashville, TN, USA;; 4 Department of Cardiovascular Science and Metabolic Diseases, Livingstone Center for Prevention and Translational Science, Livingstone, Zambia;; 5 University of Iowa, Department of Internal Medicine, Iowa City, IA, USA

**Keywords:** dynamin-related protein 1 (DRP1), exercise interventions, fission and fusion, mitochondria quality control, mitochondria–endoplasmic reticulum contact sites (MERCs), mitochondrial dynamics, mitophagy, posttranslational modifications, sarcopenia, skeletal muscle aging

## Abstract

Mitochondrial function is crucial in regulating cellular activity and determining cell fate. The replication and transcription of mitochondrial DNA are essential for maintaining mitochondrial integrity. These processes are governed by mitochondrial fission and fusion, which play a vital role in energy distribution, quality control, and metabolic regulation. Mitochondrial fission relies on the coordinated actions of mitochondria–endoplasmic reticulum contact sites, actin filaments, and dynamin-related protein 1, which collectively mediate mitochondrial constriction and fission. This interplay is fundamental to mitochondrial homeostasis and, critically, to the functionality of skeletal muscle. In this review, we explore the complex interactions among dynamin-related protein 1, mitochondria–endoplasmic reticulum contact sites, and actin and their significance for skeletal muscle function. Additionally, we discuss potential strategies to preserve these interactions, supporting optimal muscle performance in skeletal muscle aging. This review provides key insights and outlines future research directions to advance our understanding of this essential yet widely studied relationship.

## Editor note

AHJ is an Editorial Office member of Aging Advances. He is not involved in decisions about the paper which he has written himself or has been written by family members or colleagues or whoever relates to products or services in which the editor has an interest. The submission is subject to the journal’s standard procedures, with peer-review handled independently of the relevant editor and their research groups.

## Introduction

Skeletal muscle is a specialized tissue that facilitates movement, maintains posture, and generates force. Myofibrils characterize them, containing sarcomeres composed of actin and myosin filaments.^1^ Skeletal muscle function is regulated by the nervous system, relying on the synergistic interaction between motor neurons and the neuromuscular junction for efficient signal transmission.^2–4^ Additionally, skeletal muscle consists of different fiber types, including type I and II muscle fibers which are essential for specific movements and force generation.^5–7^ The physiological function of these skeletal muscle fibers depends on the frequency and intensity of physical activity and the load placed on the muscle.^1,8^ Type I fibers are fatigue-resistant due to high mitochondrial content and are primarily involved in aerobic activities.^6^ In contrast, type II fibers have lower mitochondrial content, are more susceptible to fatigue, and are engaged in anaerobic exercises.^9^ The function of these muscle fibers contributes to the overall quality of life. With aging, skeletal muscle undergoes a progressive decline in mass, strength, and function, a condition known as sarcopenia.^10,11^ This disorder leads to adverse outcomes, including mobility limitations; increased fall risk; higher mortality rates; and cardiac, respiratory, and cognitive impairments.^12–14^ The most effective treatment to date is exercise, though nutritional and pharmacological interventions are also used; however, these approaches do not fully restore muscle function.^15,16^ Therefore, there is a critical need for targeted treatments, both direct and indirect, to support muscle regeneration in individuals with sarcopenia.

## Search Strategy

We conducted a systematic literature search in PubMed, Web of Science, and Google Scholar databases covering studies from January 2000 to June 2025. We used combinations of keywords such as “skeletal muscle,” “mitochondria,” “mitochondrial quality control,” “autophagy,” “mitophagy,” “mitochondrial dynamics,” “fusion,” “fission,” “DRP1,” “OPA1,” “MERC,” “MAM,” “sarcopenia,” and “aging.” Boolean operators (AND, OR) were applied to refine the search. Inclusion criteria were: peer-reviewed original research or reviews, studies in English, and relevance to mitochondrial function in skeletal muscle or sarcopenia. Exclusion criteria included non-English articles, conference abstracts without full data, and studies unrelated to muscle or mitochondrial quality control. References from key review articles were also screened for additional relevant sources.

## Sarcopenia and Mitochondrial Energetics

Sarcopenia, a critical contributor to morbidity and mortality in older adults, is an age-associated disorder marked by the progressive weakening of skeletal muscle mass, strength, and function.^17^ Per the European Working Group on Sarcopenia in Older People, sarcopenia may be defined within three categories based on muscle mass and function: “presarcopenia,” “sarcopenia,” and “severe sarcopenia.”^18^ Clinically, it most often presents as muscle weakness that hampers an individual’s mobility, independence, and overall quality of life.^19^ Visible loss of muscle bulk, especially in the arms and legs, is a hallmark of sarcopenia.^19^ Sarcopenia can also be characterized by these additional factors, including fatigue, slower walking speed, and trouble with everyday responsibilities such as climbing stairs or rising from a chair.^15,16^ Typically, diagnosis, although varied in criteria, combines imaging-based measures of muscle mass (using DXA, MRI, or bioelectrical impedance) with assessments of power, stability (e.g., grip strength), and functional performance examinations, such as gait speed or the timed up-and-go test.^20^

People living with sarcopenia are more likely to experience falls, fractures, and even higher mortality, making it a pressing and complicated health concern.^19^ Many of these associated symptoms occur under frailty syndrome, which constitutes multisystem vulnerability with sarcopenia as a root cause.^21^ Data from the National Inpatient Sample (2014) have suggested that in the United States alone, total hospital expenditures owing to sarcopenia surpass 40 billion dollars, with nearly half of this cost coming from patients who are over 65 years of age.^22^ While the exact prevalence of sarcopenia in the United States remains contested owing to differences in diagnostic criteria, prevalence estimates range from 9%–18%, a proportion expected to rise with the aging population.^23^ While research on sarcopenia has increased dramatically over the past 20 years,^24^ there remains a need to understand its fundamental etiology and develop therapeutics.

The causes of sarcopenia are diverse and multifactorial pathogenic factors in nature.^17^ Aging itself contributes through hormonal fluctuations such as reduced levels of growth hormone and sex steroids, both of which are indispensable for sustaining muscle mass.^23,25^ Lifestyle factors play a foremost role as well; physical inactivity— especially the absence of resistance exercise— accelerates muscle loss, while poor nourishment, including low protein or vitamin D intake, worsens the decline.^16,26^ On a biological level, chronic inflammation, oxidative stress, and mitochondrial dysfunction are now understood as chief drivers.^16,27^ Recent evidence points to disruptions in mitochondrial dynamics, imbalances between fission and fusion, as a crucial connection between diminished cellular energy generation and muscle atrophy.^28^

Notably, mitochondrial functionality plays a critical role in regulating muscle volume in sarcopenia.^29^ Mitochondria are responsible for producing adenine triphosphate, which is essential for muscle contractions. However, adenosine triphosphate (ATP) production declines as sarcopenia progresses.^27,30^ Additionally, sarcopenia is associated with mitochondrial dysfunction, characterized by impaired bioenergetics, altered mitochondrial dynamics, reduced biogenesis, excessive reactive oxygen species production, defective mitophagy, and increased inflammation.^27^

## Mitochondrial Dynamics Overview

Mitochondria continuously undergo fusion and fission to maintain organelle integrity and function.^31^ Fusion refers to the merging of two mitochondria to form a larger network, facilitating the exchange of mitochondrial DNA (mtDNA), proteins, and lipids to support mitochondrial health.^32,33^ In contrast, fission involves the division of mitochondria, which is essential for removing dysfunctional mitochondria via mitophagy, and ensuring an adequate energy supply to meet cellular demands.^34^ In sarcopenia, the balance between fusion and fission is disrupted, leading to impaired muscle function.^35^ Further research is needed to develop novel therapies for mitigating the effects of sarcopenia on aging individuals.

Recent developments in organelle imaging have enabled detailed visualization and identification of organelles using methods such as transmission electron microscope and immunogold labeling.^36^ These advancements have improved our understanding of organelle-organelle interactions, especially membrane contact sites. These structures facilitate the exchange of macromolecules and metabolites, playing a crucial role in maintaining cellular homeostasis.^37–39^ Specifically, the mitochondrial fission protein dynamin-related protein 1 (DRP1) and mitochondria-endoplasmic reticulum (ER) contact sites (MERCs) have been implicated in the pathogenesis of sarcopenia.^40^ DRP1 is responsible for mitochondrial fission, and it mediates fission after its interactions with ER, actin filaments, and adaptor proteins like mitochondrial dynamics protein (MiD49/MiD51), mitochondrial fission factor (MFF), and mitochondrial fission protein 1 (FIS1) where it forms a ring leading to fission by GTP hydrolysis and whose dysfunction leads to hyper fusion of mitochondria, resulting in inefficient energy production needed for muscle function.^41–44^ Fibroblasts with defective DRP1 exhibit hyperfusion of mitochondria, aberrant cristae structure, elevated levels of glycolysis, altered mitochondrial potential, and inefficient electron transport chain coupling.^45^ Additionally, the interaction between MERCs and DRP1 is critical for calcium and lipid exchange, which supports robust muscle function.^46–49^

*Drp1* deficiency leads to dysfunctional mitochondria, ultimately triggering the ubiquitin-proteasome system and unfolded protein responses, causing changes in mitochondrial Ca^2+^ uptake.^50,51^ In tandem, this dysfunctional feedback loop leads to Ca^2+^ overload, mitochondrial swelling, and a release of factors adversely affecting myofibers.^50^ Disruptions in these contacts contribute to cellular senescence, leading to muscle degradation, highlighting MERCs and DRP1 as potential therapeutic targets for sarcopenia treatment.^52^ In sarcopenia, a decline in actin filaments leads to increased muscle degradation, impairing muscle contraction and strength.^10^ Age-related oxidative stress and inflammation further damage actin, worsening muscle loss. In subsequent sections, we will review recent evidence in understanding how DRP1, MERCs, and actin contribute to the aging process. Given the evidence of MERCs and DRP1 interactions, it is crucial to understand their impact on mtDNA separation and the role of actin in mediating these processes.

## Role of Dynamin-Related Protein 1 in Mitochondrial Dynamics, Morphology, and Aging

Mitochondrial morphology is currently characterized by eight distinct structures, which include small, large, elongated, compact, branched, donut-shaped, nanotunnel, and megamitochondria volumes.^53^ Mitochondria continuously alter their structure to optimize energy production depending on the current cellular demands. These structural changes influence the shape of cristae invaginations, which, in turn, regulate the assembly and stability of complexes involved in ATP production.^54,55^ In the context of aging, mitochondrial dynamics are disrupted due to the accumulation of adverse mutations, often compounded by a sedentary lifestyle.^35,56^ To better understand age-related skeletal muscle dysfunction, specific mitochondrial proteins should be examined for pathological changes, as they may serve as therapeutic targets for enhancing bioenergetic capacity.

**Figure 1 ｜:**
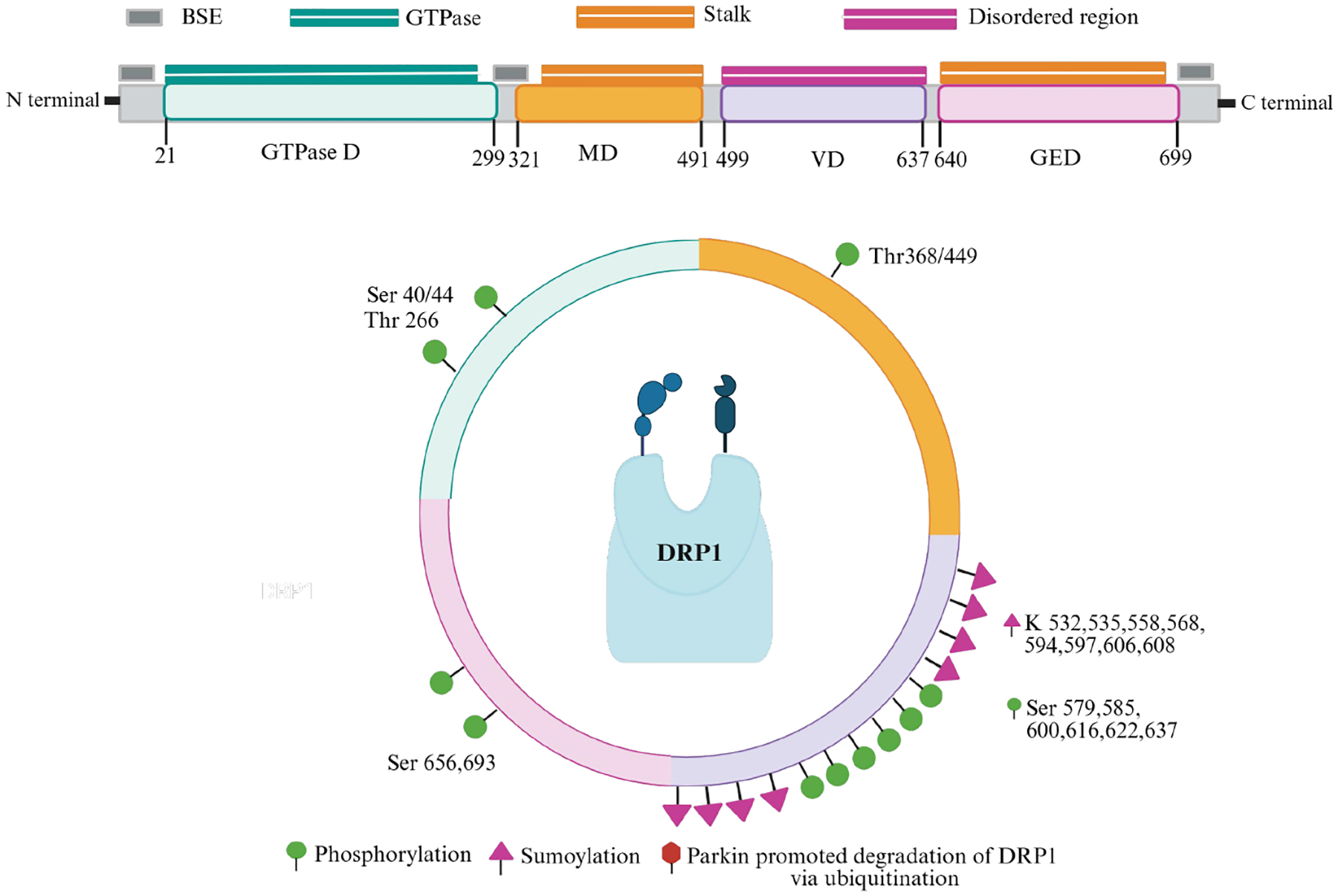
DRP1 domain architecture and post-translational modifications. Drp1 consists of four domains: GTPase domain, middle domain (MD), variable domain (VD), and GTPase effector domain (GED).^143^ The MD and GED together form the stalk and a bundle signaling element, which connects the GTPase domain to the stalk for conformational communication. DRP1 is regulated via post-translational modifications that primarily occur through phosphorylation, sumoylation, and ubiquitination modifications, which influence its activity. BSE: Bundle signaling element; DRP1: dynamin-related protein 1.

Mitochondrial fission is driven by a cytosolic GTPase known as DRP1, a member of the dynamin superfamily encoded by the *DNM1L* gene in humans.^57^ DRP1 requires oligomerization to function and is recruited by receptors such as Fis1, mitochondrial dynamics protein 49 and 51 (MiD49 and MiD51), and MFF.^58–60^ DRP1 activity is regulated through post-translational modifications, including phosphorylation, SUMOylation, S-nitrosylation, and ubiquitylation (Figure 1).^58,61–63^ Specifically, phosphorylation at Ser 616 (S616) increases DRP1 activity,^64^ promoting fission during mitosis through interactions with the mitochondrial fission factor (MFF).^65^ Once activated, DRP1 binds to its target receptor on the outer mitochondrial membrane, where it constricts around the cell, allowing dynamin 2 protein to complete the final membrane scission.

Central to phosphorylation at S616 are several players. Pink1 and cyclin-dependent kinase 8/19 in neuronal *Drosophila* play redundant roles in the promotion of the phosphorylation of Ser616.^64^ Additionally, mechanistic/mammalian target of rapamycin complex 1 (MTORC1), through the translation of mitochondrial fission process 1, results in the phosphorylation and subsequent recruitment of DRP1 (Table 1).^66^ Mechanistically, this aligns with roles in sarcopenia, as a reduction in the downstream activation targets of mTOR is observed in older rats with load-induced skeletal muscle growth.^67^ Yet, interestingly, murine liver studies show that increased mechanistic/mammalian target of rapamycin complex 1 activity diminishes cyclin-dependent kinase 8 action. Additionally, as previously reviewed,^68^ multiple other proteins have been observed to affect S616 phosphorylation, suggesting potentially altered pathways through which DRP1 is recruited to the constriction site.

**Figure 2 ｜:**
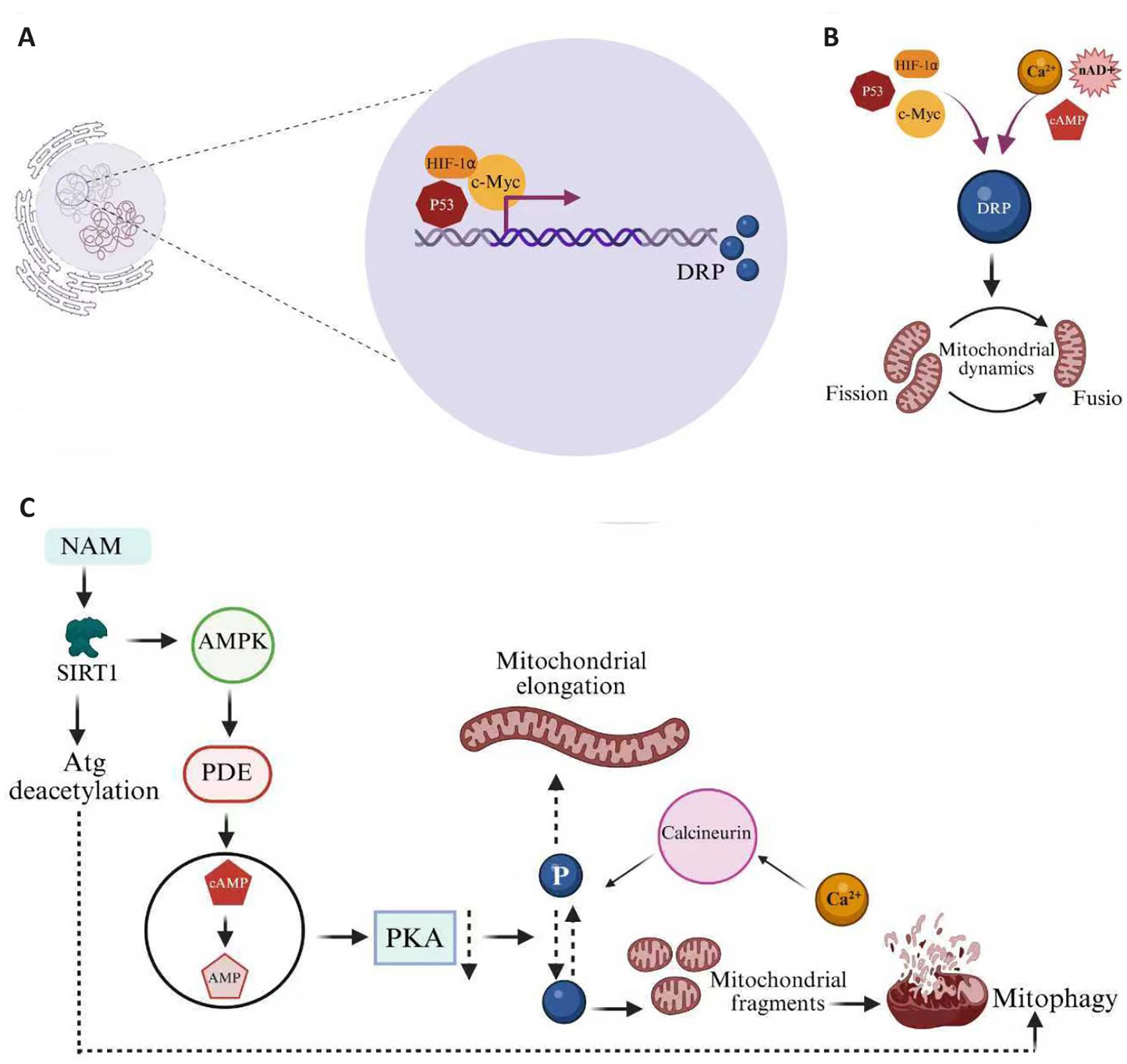
Nucleoside-mediated regulation of mitochondrial dynamics via DRP1. (A) Transcription factors p53, hypoxia-inducible factor 1-alpha (HIF1α), and c-Myc regulate DRP1 expression in response to cellular stress and metabolic demands.^144^ (B) Nuclear transcriptional programs converge with metabolic post-translational modifications to control mitochondrial fission-fusion dynamics. (C) Nucleoside-derived signals (NAM, cAMP, and Ca^2+^) directly modulate DRP1 enzymatic activity through phosphorylation and deacetylation mechanisms.^41,44^ DRP1-mediated mitochondrial dynamics represent a sophisticated regulatory network integrating nuclear transcriptional control with real-time metabolic signaling for cellular energy homeostasis. AMPK: AMP-activated protein kinase; DRP1: dynamin-related protein 1; NAM: nicotinamide; PDE: phosphodiesterase; PKA: protein kinase A; SIRT: sirtuin.

This fission process is critical for maintaining the balance between fusion and fission, which is essential for cellular homeostasis and bioenergetic capacity. In aged murine skeletal muscle, both *Drp1* knockdown and *Drp1* overexpression result in skeletal muscle atrophy concomitant with impaired autophagy and redox state.^69^ DRP1 also plays a key role in regulating mitophagy by isolating damaged mitochondria for selective degradation (Figure 2).^65^ Additionally, DRP1 facilitates in the release of cytochrome-c from mitochondria, a crucial step in activating the programmed cell death pathway. DRP1 can also be self-activated in response to oxidative stress conditions, leading to mitochondrial fragmentation.^68^ However, excessive reactive oxygen species production can also lead to overactivation of DRP1, resulting in excessive mitochondrial fragmentation, contributing to cellular damage and dysfunction.^70–72^

Hypoxia is one such mechanism that may interconnect DRP1 with sarcopenia. Counterintuitively, hypoxia generates reactive oxygen species through mechanisms that remain poorly elucidated but are generally understood to involve insufficient amounts of electron acceptors in the electron transport chain.^73^ In hypoxia, a hypoxia-inducible factor 1-alpha-mediated mechanism causes a glycolytic shift towards type II fibers,^74^ while hypoxia-inducible factor 2-alpha stabilization diminishes muscle stem cells proliferation.^75^ In turn, in crustaceans under hypoxia, hypoxia-inducible factor 1-alpha promotes *Drp1* in an MTP18-dependent mechanism to cause excess mitochondrial fission.^76^ Owing to these findings and similarities between hypoxia and aging, past commentaries have advocated for the usage of hypoxic models for the study of sarcopenia.^77^ Newer findings from the Caudwell Xtreme Everest Expedition further show that hypoxia can prompt the development of sarcopenia.^78^ These findings have been recapitulated in a sleep study, showing that patients with chronic obstructive pulmonary disease displaying nocturnal hypoxemia exhibited exacerbated sarcopenia development.^79^ Pertinently, disuse atrophy differs by muscle type^80^ and fiber-dependent shifts to Type II via hypoxia suggest one mechanism by which relative fiber distribution affects susceptibility to sarcopenia. Beyond the therapeutic potential to prevent nocturnal hypoxemia to protect against sarcopenia,^81^ this underscores an unexplored modulatory link between DRP1 and sarcopenia.

Outside hypoxia, a previous study investigated the role of DPR1 in skeletal muscle metabolism, particularly its impact on mitochondrial function and complex assembly. The study found that DRP1 is essential for maintaining fatty acid oxidation and insulin sensitivity in muscle.^82^ Mutations in *Drp1* potentially impact fatty acid oxidation, influencing the peroxisomal morphology. The inhibition of mitofusin (MFN)2 can have a positive impact on the elongation of peroxisomes independently of the mitochondrial network, suggesting the regulation of MFN2 via activators and inhibitors to determine the role of peroxisomes to the metabolic defects in EMPF1 encephalopathy patients.^45^ Knockdown of *Drp1* in mice led to mitochondrial hyperfusion, impaired lipid metabolism, and accumulation of succinate, indicating a disruption in complex II activity. Since complex II is a key contributor to muscle fatigue and atrophy, its dysfunction has significant implications. The study further identified that these impairments were linked to reduced mitochondrial translocation of succinate dehydrogenase complex assembly factor 2 (SDHAF2), a crucial assembly factor for complex II. Notably, restoring *Sdhaf2* expression in *Drp1*-knockdown myocytes rescued complex II function, improving fatty acid oxidation and insulin signaling.^83^ Mutant cells also tend to have a hyperpolarized nature due to Fo F1 ATPase function, inhibition of complex 1, as well as voltage-dependent anion channel (VDAC) opening. Glucose depletion within the cells, as a part of elevated glycolysis, can also cause the membrane potential to be hyperpolarized. Abnormal fission is also found to affect pyrimidine synthesis and dihydroorotate dehydrogenase activity.^45^ These findings establish a direct link between mitochondrial morphology, metabolic regulation, and skeletal muscle health, suggesting that targeting DRP1-SDHAF2 interactions could offer new therapeutic strategies for age-related skeletal muscle disorders.

DRP1 is emerging as a key marker of dysfunctional muscle conditions such as sarcopenia, myopathy, amyotrophic lateral sclerosis, encephalopathy,^45^ and Duchenne muscular dystrophy.^83^ This raises the question of which mitochondrial proteins respond to aerobic *versus* anaerobic exercise. While most studies focus on aerobic training, incorporating anaerobic training is crucial to determine the optimal exercise modality for restoring mitochondrial function. Additionally, cellular-level studies are needed to clarify these discrepancies and provide deeper insights into the role of exercise in aging.

Recently, Gonzalez et al.^84^ have highlighted that age-dependent sarcopenia should not only be defined by loss of skeletal mass, but also displays hallmarks including type II (fast-twitch) fiber atrophy and neuromuscular decline. Since sarcopenia still lacks a consensus definition, metrics have been proposed to diagnose on inclusion criteria such as the number of neuromuscular junctions.^85^ Indeed, this aligns with recent conceptions of the brain–body energy conservation model of aging, wherein brain biochemistry modulates physical inactivity.^86^ As previously reviewed,^87^ mitochondrial bioenergetic defects in neuromuscular junctions may contribute to sarcopenia. Since dysregulated Drp1 activity is a hallmark of many neuronal pathologies,^88^ such interdependences in the context of sarcopenia are worth further investigating.

Recent data have revealed significant controversies regarding DRP1 regulation in aging. Some studies report that increased DRP1 expression and mitochondrial fragmentation with age, suggesting overactive fission as a driver of muscle decline. For example, Halling et al.^89^ found that aging increased DRP1 content relative to mitochondrial mass, correlating with fragmented networks. In contrast, other evidence suggests that insufficient DRP1 activity can accumulate dysfunctional mitochondria. Dulac et al.^69^ showed that both DRP1 overexpression and knockdown in late middle-aged mice caused muscle atrophy and mitochondrial dysfunction, indicating that maintaining DRP1 within a narrow range is critical. These conflicting data imply that both excessive and deficient fission may be detrimental. In *Drosophila*, augmenting *Drp1* improved muscle aging, whereas in mice, DRP1 manipulation harmed muscle integrity.^69^ Thus, the optimal DRP1 level in aged muscle remains unclear, underscoring a complex balance between fission and fusion in sarcopenia (Table 2).

## Mechanisms and Pathways

In the context of mitochondrial quality control, the major pathways include proteostasis, biogenesis, dynamics (fusion/fission), and autophagy/mitophagy.^90^ Fusion proteins (MFN1/2 and OPA1) oppose fission proteins (DRP1 and FIS1), and their balance maintains network morphology.^41^ Excessive DRP1 activity can fragment mitochondria, potentially triggering apoptosis, whereas insufficient DRP1 may hinder removal of damaged mitochondria. The mechanistic underpinnings of these effects are still being resolved; one possibility is that too much fission causes energy loss, while too little allows dysfunctional organelles to persist.

Table 3 summarizes key mitochondrial quality control mechanisms and associated pathways discussed in this review. Table 4 outlines major technologies used to study mitochondrial and cellular stability in muscle. These tables classify and condense the mechanisms and tools for easy reference.

## Interplay Between Mitochondria–Endoplasmic Reticulum Contact Sites and Dynamin-Related Protein 1

The largest organelle in the cell is the ER, a continuous network that facilitates protein transport, synthesis, folding, lipid, and calcium exchange to maintain homeostasis. In response to inflammation, the ER can trigger a stress signal, leading to the unfolded protein response to restore balance.^91^ The altered ER chaperons calnexin, Bip/GRP78, and the downregulation of MFN2 are some specific changes observed in the loss-of-function approach of *Drp1*. The ER stress and unfolded protein response activation induced high levels of FGF21, impacting different phenotypes in knock-out mice, where it caused regeneration and muscle degeneration in *Drp1* knock-out.^51,92^ Left unchecked, prolonged ER stress can result in apoptotic events.^93^ Additionally, the ER interacts with various organelles, including the nucleus, ribosomes, Golgi apparatus, lysosomes, plasma membrane, peroxisomes, and mitochondria, serving as a key regulator of intracellular communication and homeostasis.^37^

**Figure 3 ｜:**
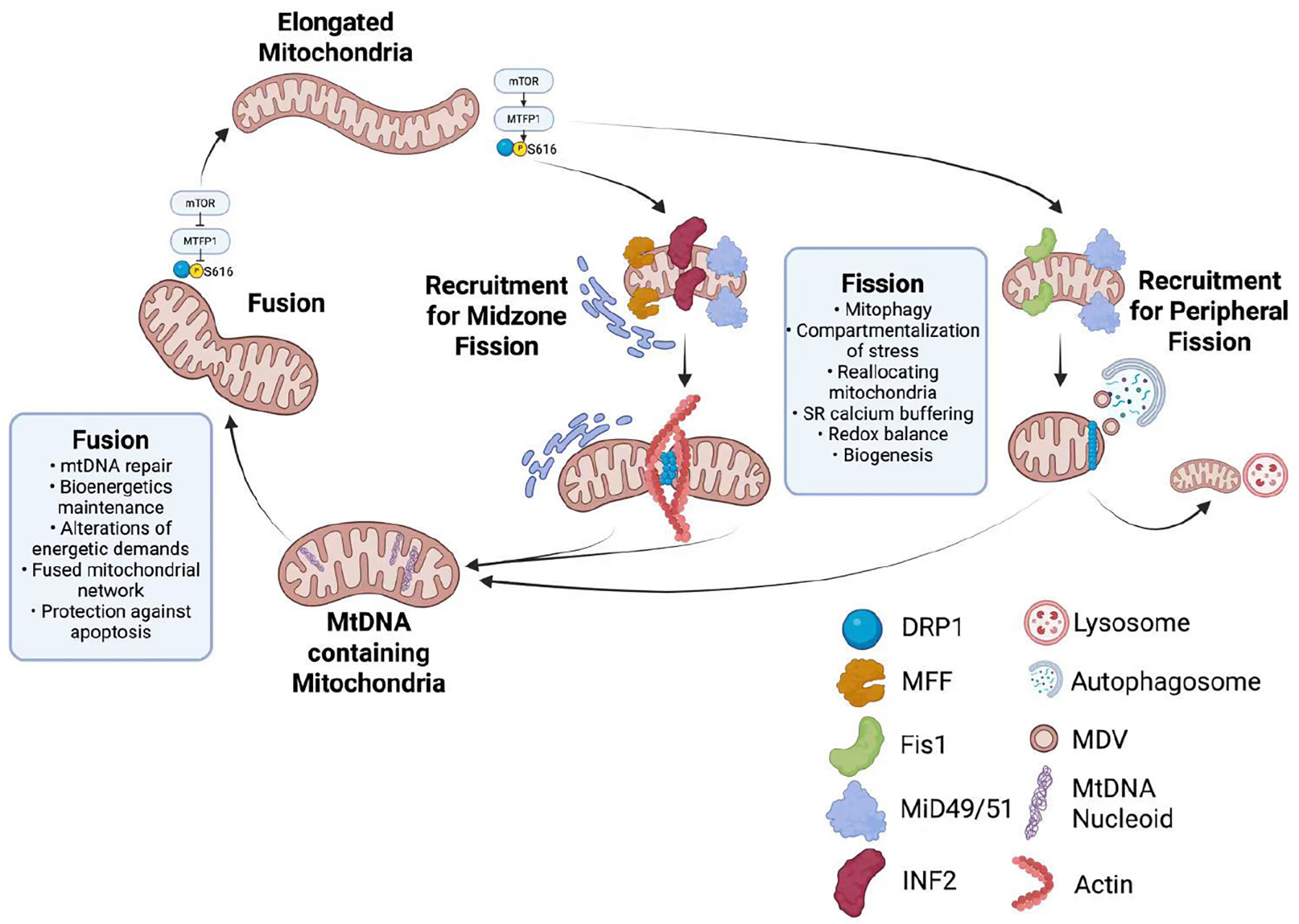
Mitochondrial dynamics and dual fates of DRP1-mediated fission. Mitochondria undergo a complex process of fusion and fission.^66^ In cases of elongated mitochondria, mechanistic (or mammalian) target of rapamycin complex 1 (MTORC1) promotes the translation of mitochondrial fission process 1 (MTFP1), thereby influencing dynamin-related protein 1 (DRP1) phosphorylation at serine 616 and 637.^96^ In mitochondrial fission factor (MFF)-mediated midzone fission, actin is recruited through inverted formin-2 (INF2), and ER contacts are formed, generating daughter mitochondria containing nucleoids. In mitochondrial fission 1 protein (Fis1)-mediated peripheral fission, typically, autophagosomes are recruited and mitochondrial-derived vesicles are released (MDVs). Some small daughter cells may not contain nucleoids, in turn typically forming lyososmal contact sites.^94,96^

MERCs play a crucial role in cellular health, as both organelles undergo structural and functional changes depending on cellular demands.^94^ MERCs are responsible for calcium exchange, transferring calcium (Ca^2+^) from the ER to mitochondria—an essential process, as Ca^2+^ facilitates DRP1 activation for mitochondrial fission (Figures 2 and 3).^95^ Indeed, the presence of MERCs, alongside either MFF or FIS1, can be a determinant of either midpoint or peripheral fission occurring, which affects daughter mitochondrial fates.^96^ Additionally, MERCs contribute to lipid transfer, autophagy, and immune responses; while some findings suggest that MERCs may also promote premature cellular senescence.^97^ Specifically, research on the inositol 1,4,5-trisphosphate receptor type 2 calcium channel revealed that the absence of inositol 1,4,5-trisphosphate receptor type 2 extends lifespan and reduces cellular senescence.^40^ However, these findings were derived from studies using MRC-5 cells from 14-week-old fetuses,^40^ highlighting the need to investigate MERC function in aging, particularly within aged skeletal muscle. However, the causal role of MERC dysfunction in sarcopenia is still emerging. Aging is accompanied by altered MERC ultrastructure and protein composition,^98^ and these changes are associated with slower muscle relaxation and early contractile deficits. Allen et al. reported that skeletal muscle from older mice shows disrupted MERC morphology and altered levels of MERC-associated proteins, linking these changes to early muscle aging.^98^ These data suggest MERCs may be involved in sarcopenia onset. Yet, direct evidence that MERC disruption alone drives sarcopenia is limited; most findings are correlative.

MERCs interact with DRP1 in several ways. In mid-point fission, MERCs are dependent on the recruitment of DRP1 to the mitochondrial constriction site, alongside actin.^96^ Conversely, DRP1 is recruited to MERCs through the outer mitochondrial membrane protein FUNDC1, propelling mitophagy under hypoxia,^99,100^ which may be associated with autophagic processes often associated with peripheral fission.^96^ It is also well understood that in high-glucose conditions, increased MERC formation leads to A-kinase anchoring protein 1-DRP1 interactions, triggering excessive fission.^101^

While MERC and DRP1 interactions have been reasonably elucidated, one area of uncertainty remains the influence of calcium. Within adult murine cardiomyocytes, it is understood that DRP1 is recruited to the mitochondria-SR associations through mechanisms likely involving calcium signaling.^102^ In methylmercury-induced neurotoxicity, this was mechanistically explained via mitochondrial calcium influx, forming a positive feedback loop in which AMPK-driven DRP1-mediated fission enhanced MERC formation and further calcium transfer.^103^ DRP1-mediated fission in skeletal muscle has also been shown to be necessary in normal MERC formation and calcium homeostasis. Favaro et al.^104^ have shown that in its absence, mitochondrial calcium uptake via mitochondrial calcium uniporter (MCU) activates an activating transcription factor 4 (ATF4)-dependent stress signaling, leading to ER stress. Increased ATF4 expression is known to induce skeletal muscle fiber atrophy.^26^ We have also found MERC tethering occurs in ATF4-dependent mechanisms in skeletal muscle following OPA1 deletion.^105^ ATF4 deletion in a murine model also conferred protection against age-related sarcopenic phenotypes.^106^ Other studies have shown that ATF4 overexpression decreases the levels of DRP1,^107^ while *Drp1* expression is inversely linked with the overexpression of ATF4, leading to muscle atrophy.^108^ Together, this establishes a complex and under-researched axis involving DRP1-ATF4, calcium homeostasis, and MERC formation, which contributes to skeletal muscle atrophy.

Several important questions still remain for MERCs and DRP1. While neuromuscular junction instability has been related to ER stress in aging skeletal muscle,^109^ it remains unclear how neuronal DRP1-dependent MERC changes are connected with sarcopenia. Similarly, the importance of relative rates of midzone and peripheral fission remains unclear. While MERCs are only involved in the former, the latter has been shown to promote mitochondria-lysosome contacts.^99^ Lysosomal protein degradation supports muscle integrity, and both low and high autophagy contribute to age-related sarcopenia.^110^ Thus, it is plausible that a misbalance of pro-autophagic fission (peripheral) and MERC-involved fission (midpoint) may contribute to sarcopenia. Finally, further mechanistic studies are needed to establish whether manipulating MERC components (e.g., IP3R-VDAC complexes) can influence DRP1 and muscle aging outcomes.

### Differential effects of exercise modalities on dynamin-related protein 1 and mitofusin 2

Exercise intensity and type distinctly influence skeletal muscle expression of DRP1 (fission) and MFN2 (fusion). For example, high-intensity interval training robustly upregulates both MFN2 and DRP1 protein levels in human skeletal muscle.^111^ Likewise, chronic resistance training (~10 weeks) markedly increases MFN1, MFN2, and OPA1 (fusion) and DRP1 in older adults’ muscle.^112^ By contrast, a physically active (endurance-trained) lifestyle elevates fusion markers (*Mfn2* and OPA1) compared to sedentary aging.^113^ Notably, one study found that aerobic (moderate) exercise reduced DRP1 activation (Ser616 phosphorylation) while raising fusion gene expression (*Opa1* and *Mfn1/2*) and fat oxidation,^114^ suggesting that higher-intensity or endurance exercise favors a more fused, oxidative phenotype. In summary, intense endurance or combined training protocols tend to strongly induce MFN2 (and often DRP1) to remodel mitochondria,^111,112^ whereas lower-intensity or resistance-only programs also enhance fusion but may yield smaller or variable changes in DRP1.

## Recent Advances: SUMOylation of DRP1 and Mitochondria–Endoplasmic Reticulum Contact Site Remodeling in Aging and Exercise

Recent work has revealed that SUMOylation critically governs DRP1-dependent fission in muscle. SUMO1 conjugation to DRP1 stabilizes the protein and promotes its persistent association with the outer mitochondrial membrane, thereby enhancing mitochondrial fission.^115^ In contrast, DRP1 modified by SUMO2/3 is less efficiently recruited to mitochondria.^115^ The balance of SUMOylation is dynamically set by the SENP proteases: for example, SENP5 removes SUMO from DRP1 and suppresses fission, yielding enlarged, hyperfused mitochondria.^41^ Intriguingly, DRP1 phosphorylation and SUMOylation cooperate to fine-tune activity: a non-phosphorylatable DRP1 (S616A) becomes hyper-SUMOylated, whereas a constitutively phosphorylated mimic is hypo-SUMOylated.^115^ Conversely, preventing DRP1 SUMO conjugation increases its Ser616 phosphorylation.^115^ Thus, SUMO1/2 ligation (and removal) acts as a gate for DRP1’s mitochondrial targeting and oligomerization. These modifications in turn dictate mitophagy: persistent DRP1-SUMO1 triggers clearance of damaged mitochondria,^41^ whereas excessive deSUMOylation (as in SENP5 overexpression) blunts fission and autophagy.^41^

Although direct *in vivo* studies in aged muscle are still needed, these mechanisms imply that aging could perturb DRP1 SUMOylation and thereby alter mitochondrial network morphology. For instance, if aging muscle upregulates SENP5 (as seen in failing heart), DRP1 would lose SUMO1 and fission could decline, resulting in enlarged, dysfunctional mitochondria.^41^ Conversely, failure to deSUMOylate DRP1 (or a shift toward SUMO1 dominance) might over-activate fission and mitophagy.^41^ Such an age-dependent drift in the SUMO1/SUMO2 balance would be expected to affect both mitochondrial turnover and network architecture during Sarcopenia. In sum, recent in vivo findings underscore a complex post-translational circuit by which SUMO1/2 modifications regulate DRP1 stability, localization and activity in muscle, with implications for age-related changes in mitophagy and mitochondrial morphology.^41^

Concurrently, new evidence shows that endurance exercise remodels MERCs in aging muscle, preserving Ca^2+^ handling and function. High-resolution transmission electron microscope of skeletal muscle in middle-aged mice (~18–24 months) revealed that early age-related muscle dysfunction is accompanied by shorter MERC lengths and reduced contact coverage, which likely slows Ca^2+^ transfer between SR and mitochondria.^98^ Critically, 6–8 weeks of regular endurance training prevented these ultrastructural defects: aged trained mice maintained normal MERC geometry and faster relaxation kinetics.^98^ At the same time, proteomic analysis of isolated muscle MAM fractions showed that exercise reverses aging-associated declines in key MAM proteins (including Ca^2+^-transporters and metabolic regulators).^98^ In functional terms, preserving MERCs is expected to sustain ER to mitochondria Ca^2+^ flux. For example, the Bcl-2 family protein BCL2L13, when elevated by endurance training, localizes to ER-mitochondria junctions and supports Ca^2+^ signaling: loss of BCL2L13 (in zebrafish or C2C12 myotubes) blunts mitochondrial Ca^2+^ uptake and impairs muscle performance.^116^ In this way, exercise-induced MERC remodeling (both structural and molecular) enhances Ca^2+^ handling and bioenergetic coupling, helping to mitigate sarcopenia-related muscle decline by preserving excitation–contraction efficiency and mitochondrial quality.^98,116^

### Mitochondria–endoplasmic reticulum contact sites modulating mitochondrial DNA mutation and segregation risk

Mitochondria are unique among organelles because they contain their own DNA, known as mtDNA, which encodes essential proteins for oxidative phosphorylation and ATP production.^117^ Multiple copies of the mitochondrial genome are packaged in nucleoid, large nucleoprotein complexes, and are then distributed into mitochondria, which occurs through fusion and fission.^118,119^ Interestingly, while both midpoint and peripheral fission exhibit nucleoid transfer to daughter mitochondria, only peripheral fission exhibits cases in which nucleoids are not transferred to a daughter mitochondrion.^96^ Unlike nuclear DNA, mtDNA lacks robust repair mechanisms, making it highly susceptible to mutations caused by oxidative stress and environmental factors. Mitochondrial transcription factor A (TFAM) getting depleted can also lead to a reduction in mtDNA integrity and inflammatory mtDNA releases into the cytosol as it is crucial for replication initiation.^118^ Additionally, mtDNA mutations can be maternally inherited. High mutation loads disrupt oxidative phosphorylation, impair ATP production, and create metabolic imbalances, particularly affecting high-energy-demanding tissues such as skeletal muscle.^119^ Research has linked mtDNA mutations to apoptotic events and sarcopenia in mitochondrial-mutant mice. The main polymerase involved in generating RNA primers at the replication origins is POLRMT, a sole polymerase required for mtDNA transcription, and the DNA synthesis is carried out by DNA polymerase gamma holoenzyme.^118^ Specifically, a study utilizing a mouse model with a deficient version of mtDNA polymerase γ, an enzyme responsible for mtDNA replication and repair, found increased random mtDNA mutations.^120,121^ Gene expression profiling revealed downregulation of mitochondrial function-related genes, reduced electron transport chain complex content, lower respiration, decreased ATP levels, and reduced mitochondrial membrane potential.^120^

Emerging evidence suggests that MERCs play a role in mtDNA synthesis and nucleoid distribution during mitochondrial division.^31,122,123^ Researchers used TFAM tagged with GFP to label polymerase γ, followed by pulse-labeling with 5-ethynyl-2’-deoxyuridine to track newly synthesized mtDNA. Time-lapse imaging revealed that actively replicating nucleoids were selectively positioned at MERCs destined for mitochondrial division.^124^ These findings suggest that MERCs function as mediators of proper mtDNA segregation, potentially influencing mutation rates and nucleoid distribution. Understanding MERC involvement in age-related mtDNA mutations and nucleoid distribution in skeletal muscle may help optimize muscle performance.

## Actin Dynamics in Cellular Aging

Actin is an abundant cytoskeletal protein that exists in two forms: globular actin and filamentous actin (F-actin).^125,126^ In skeletal muscle, actin forms thin filaments within sarcomeres, where it interacts with myosin to facilitate contraction. Additionally, actin plays a crucial role in cellular structure maintenance, mechanotransduction, and intracellular transport.^125,126^ Actin dynamics are regulated by actin-binding proteins such as cofilin, tropomyosin, and profilin, ensuring filament stability and muscle function. This balance is essential for force generation, transmission, and muscle adaptability. However, as aging progresses, actin dynamics become disrupted, leading to functional impairments.^127,128^

Aging-associated changes in actin dynamics contribute to sarcopenia, characterized by impaired actin polymerization due to oxidative stress, post-translational modifications, calcium signaling disruptions, mitochondrial dysfunction, and actin-binding protein dysregulation.^129,130^ These impairments causecy to skele ta l disorganization, reducing muscle adaptability to mechanical stimuli. A previous study examined actin cytoskeletal dynamics in brain aging and found that F-actin accumulation disrupts cellular homeostasis, impairs autophagy, and contributes to cognitive decline and reduced health span.^131^ These findings suggest that excessive actin polymerization and cytoskeletal disorganization could be potential therapeutic targets for age-related cognitive decline.^131^

Key actin regulators undergo complex control that can be disrupted with muscle aging:

### Formins

Formin proteins (e.g., FHOD3) nucleate and elongate linear F-actin via their conserved FH1/FH2 domains.^132^ FH2 dimers initiate new filaments and remain progressively on the barbed end, while FH1 binds profilin-actin to fuel rapid elongation.^132^ In striated muscle, FHOD-family formins localize to Z-disks and are essential for sarcomere integrity; for example, FHOD3’s actin-elongation activity is required for proper myofibril assembly in muscle cells.^132^ Formins are typically autoinhibited and are activated by Rho-family GTPases (e.g., RhoA) – releasing inhibition to trigger polymerization. Dysregulation or age-related decline in formin activity could thus impair sarcomeric maintenance and repair, contributing to contractile dysfunction (though specific aging studies in skeletal muscle are still emerging).

### Arp2/3 complex

The Arp2/3 complex nucleates branched actin networks (at ~70° angles) and is activated by nucleation-promoting factors such as WASP/WAVE downstream of Rac/Cdc42 signaling. In muscle cells, Arp2/3-driven polymerization builds dynamic cortical networks. For example, insulin-stimulated GLUT4 translocation in myoblasts requires Rac-dependent Arp2/3-mediated actin polymerization beneath the membrane.^133^ Arp2/3 and cofilin work in concert: Arp2/3 generates new branches while cofilin later severs filaments, enabling a cycle of rapid turnover.^133^ In aging muscle, if Arp2/3 activity or its regulation by upstream signals declines, this could compromise adaptive cytoskeletal remodeling (e.g., during repair or hypertrophy).

### Cofilin

Cofilin binds F-actin and severs filaments, increasing actin dynamics. Its activity is tightly controlled by phosphorylation: active (unphosphorylated) cofilin severs actin, whereas phosphorylation on Ser3 by LIM kinase (LIMK, downstream of RhoA/ROCK or Rac/PAK pathways) inactivates cofilin.^134^ Cofilin is reactivated by cofilin-specific phosphatases (SSH family), which dephosphorylate it.^134^ In muscle atrophy models, cofilin tends to become dephosphorylated (activated) (e.g., 48–72 hours of serum-starvation in L6 myotubes led to a marked decrease in phospho-cofilin),^134^ suggesting enhanced F-actin severing during catabolic stress. Chronic stress or aging could dysregulate this balance (for instance, excessive active cofilin can lead to cofilin-actin rod formation), potentially disrupting cytoskeletal integrity and contractile function in aging fibers.

## Mitochondria and Endoplasmic Reticulum-associated Actin Contribute to Mitochondrial Dynamics

Since MERCs regulate calcium signaling, lipid transfer, and mitochondrial dynamics, recent research has investigated the role of actin in mitochondrial fusion and fission.^135^ Live-cell imaging studies demonstrated that actin polymerization occurs on both mitochondria and the ER and is essential for mitochondrial fusion. Researchers identified distinct mitochondrial fusion sites, revealing that actin filaments function as “bridges” between fusing mitochondria. They observed two primary types of fusion events: tip-to-side fusion (where the tip of one mitochondrion fuses with the side of another) and tip-to-tip fusion (where the tips of two mitochondria fuse).^135^ Disruption of actin polymerization on mitochondria prevented fusion, negatively impacting skeletal muscle function.

While actin’s role in mitochondrial fission is well established–facilitating division by recruiting the fission protein DRP1^136,137^ –this study provides new evidence of actin’s contribution to mitochondrial fusion. Understanding how actin dynamics change with aging and how actin interacts with the ER and mitochondria could inform potential therapeutic strategies for age-related mitochondrial dysfunction.

## Pharmacological Dynamin-Related Protein 1 Modulators, Lifestyle Interventions, and Gene Therapy Approaches

Several strategies targeting mitochondrial dynamics show promise in preclinical studies:

### Drp1 inhibitors (Mdivi-1 and analogs)

Mdivi-1 (a quinazolinone compound) is widely used to inhibit Drp1-mediated fission. It has been shown to counteract pathological mitochondrial fragmentation and dysfunction. For example, in muscle cells and patient fibroblasts lacking OPA1, Mdivi-1 reduced DRP1 phosphorylation and mitochondrial fragmentation while normalizing mitophagy.^138^ In obese/diabetic mouse models, Mdivi-1 restored fused mitochondrial networks, decreased reactive oxygen species, and markedly improved insulin-stimulated glucose uptake.^115^ These findings indicate that DRP1 inhibition can rescue mitochondrial defects, though Mdivi-1’s off-target complex-I inhibition highlights the need for more specific agents.^114,138^

### Peptide inhibitors

Short peptides designed to disrupt DRP1’s interactions offer high specificity. P110 is a 7–amino-acid peptide that selectively blocks Drp1 binding to its adaptor Fis1 (without affecting other adaptors). In neuronal and muscle cell models of Alzheimer’s disease, P110 prevented amyloid β-induced DRP1 recruitment and mitochondrial fragmentation. Importantly, chronic P110 treatment in AD transgenic mice improved cognitive deficits and reduced amyloidβ pathology and oxidative stress.^139^ These results demonstrate that targeted DRP1-Fis1 inhibition can be neuroprotective and may translate to muscle aging contexts where excessive fission is detrimental.

### Lifestyle interventions

Exercise itself acts as a “mitochondrial medicine.” As noted above, aerobic exercise training in humans reduced active DRP1 (Ser616) levels and upregulated fusion genes, shifting muscle toward a fused network.^114^ Such training improved fat oxidation and insulin sensitivity, suggesting systemic metabolic benefits via altered mitochondrial dynamics.^114^ Similarly, caloric restriction or intermittent fasting (not shown here) are known to enhance mitochondrial fusion capacity. Regular physical activity thus functions as a physiological DRP1 modulator – reducing fission signaling and boosting fusion (MFN2) in aging muscle.

### Mitofusin activation and gene therapy

Strategies to enhance fusion are also promising. Small-molecule mitofusin activators (allosteric ligands that favor the fusion-competent conformation of MFN2) have reversed disease phenotypes in models of Charcot-Marie-Tooth type 2A (MFN2 mutation).^140^ In those murine studies, intermittent or sustained dosing restored mitochondrial motility and halted neurodegeneration, even in aged animals.^140^ Gene therapy approaches are likewise under study: for instance, viral-mediated OPA1 or MFN2 overexpression can correct mitochondrial morphology in patient-derived cells and animal models of optic atrophy or neuropathy.^138,140^ These interventions show that directly boosting fusion machinery can improve muscle cell health.

### Preclinical efficacy and translational potential

Most of these interventions remain at the experimental stage. Mdivi-1 and P110 demonstrate clear benefit in cell and animal models of metabolic, cardiac, and neurodegenerative disease,^114,139^ but human trials are lacking. Similarly, mitofusin activators have been tested in rodents (e.g., CMT2A) with success.^140^ Translating these findings will require careful validation of safety and specificity. However, expectations of all these inhibitors should be tempered by several recent findings, which show the narrow spectrum of physiologically viable *Drp1* ranges in sarcopenia, and that overexpression or knockout of *Drp1* typically contributes to aggravated sarcopenic phenotypes.^69,141^ Still, modulating DRP1/MFN2 shows strong preclinical efficacy and represents an active area for future translational research.^114,140^

## Limitations

Several limitations constrain the conclusions of this review. First, much of the discussed literature relies on animal or *in vitro* models, which may not fully recapitulate human aging. For example, findings in rodents or flies (such as the benefits of altered DRP1) may not translate to humans.^69^ Human muscle data on mitochondrial dynamics and MERCs are scarce. Exacerbating this issue is that *in situ* energetic deficits that occur with aging are exaggerated in isolated mitochondrial models, commonly used.^142^ Second, many studies provide descriptive correlations (e.g., changes in protein levels) without probing underlying mechanisms. Consequently, some proposed links (e.g., MERC alterations causing sarcopenia) remain speculative. Lastly, variations in methodology (different assays for mitophagy, or mitochondrial metrics) make direct comparisons difficult. We highlight these limitations so readers can interpret this review with appropriate caution.

## Conclusion

Skeletal muscle function is crucial for movement, posture, and force generation, relying on complex interactions between muscle fibers, mitochondria, and the nervous system. Aging disrupts these crucial processes, leading to sarcopenia, characterized by muscle loss, mitochondrial dysfunction, and impaired bioenergetics. Key regulators such as DRP1 and MERCs influence mitochondrial dynamics, with disruptions contributing to cellular senescence and muscle degradation. Actin dynamics also play a critical role in maintaining mitochondrial and cytoskeletal integrity, further impacting muscle function. Recent advancements in organelle imaging and molecular research highlight the need for targeted therapies to restore mitochondrial and skeletal muscle health in aging individuals. Addressing these mechanisms could offer promising therapeutic strategies to combat age-related muscle decline.

## Figures and Tables

**Figure 4 ｜:**
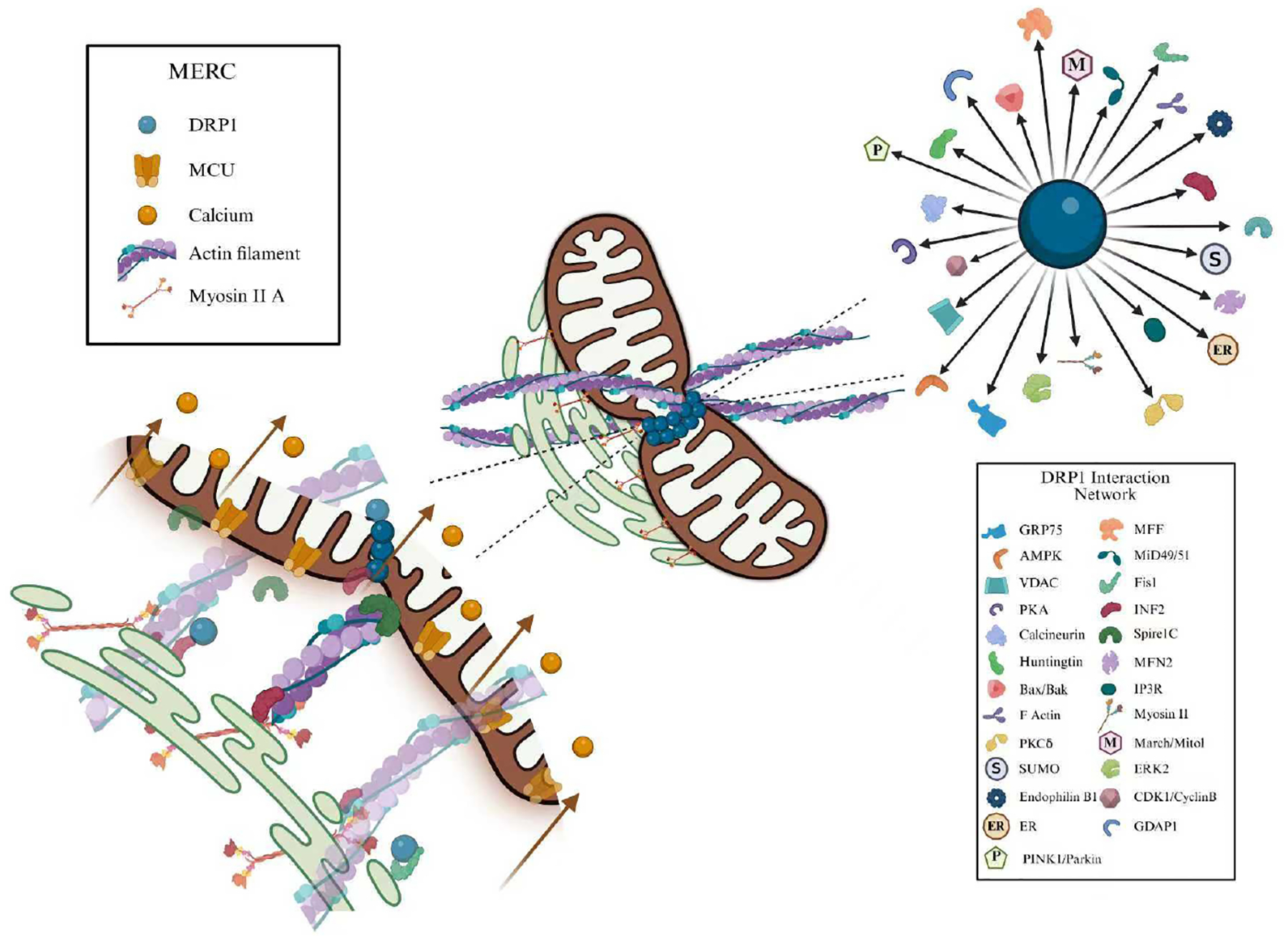
DRP interaction network at MERCs. The central DRP molecule interacts with a number of proteins forming a network, including mitochondrial adaptors (MFF, MiD49/50, Fis1), endoplasmic reticulum (ER)-associated proteins (INF2, Spire1c, and Myosin II), ER-Mito tethering complex (MFN2, IP3R, GRP75, VDAC), regulatory signaling proteins (AMPK, PKA, calcineurin), post-translational modulators MARCH/MITOL and SUMO, cell cycle regulator CDK1/cyclin B, signaling modulators PKC delta, ERK2, mitophagy regulators PINK1/Parkin and disease-related interactors (Huntingtin, Bax/Bak). Mitochondrial fission is facilitated by the cytoskeleton components.^94^ The ER elongates and encircles the mitochondria at contact sites, mediated by various proteins, like INF2 at the ER membrane, Spire1C in the mitochondrial outer membrane, and mitochondrial calcium uniporter. DRP1: Dynamin-related protein 1; ERK2: extracellular signal-regulated kinase 2; GDAP1: ganglioside-induced differentiation-associated protein 1; MERCs: mitochondria-endoplasmic reticulum contact sites; MFF: mitochondrial fission factor; MFN: mitofusin; PINK1: PTEN-induced kinase 1; PKC delta: protein kinase C delta; VDAC: voltage-dependent anion channel.

**Table 1 |:** Summary of mTOR involvement in DRP1 recruitment

Condition	MTFP1	DRP1 phosphorylation (S616)	DRP1 recruitment	Mitochondrial morphology
High Mtorc1 activity	↑	↑ (pro-fission)	High	Fragmented, punctate mitochondria
mTOR inhibition (active-site)	↓	↓	Low	Hyperfused, branched networks
mTOR inhibition + MTFP1 rescue	↑	↑	Restored	Fragmented

Data are sourced from Morita et al.^66^ DRP1: Dynamin-related protein 1; MTFP1: mitochondrial fission process 1; mTOR: mechanistic (or mammalian) target of rapamycin; MTORC1: mechanistic (or mammalian) target of rapamycin complex 1.

**Table 2 |:** Comparison of DRP1 regulation controversies in aging skeletal muscle

Controversy	Excessive DRP1 (Hyper-fission)	Insufficient DRP1 (Hypo-fission)
Observations	Increased mitochondrial fragmentation; elevated DRP1 expression in aged muscle.^89^	Elongated, clustered mitochondria with age; reports of reduced DRP1 levels in sarcopenia.
Evidence	Some studies (mouse models) link high DRP1 with apoptosis and muscle loss.^69^	Studies (mice) show *Drp1* knockdown causes severe atrophy and dysfunction.^69^
Muscle effects	Mitochondrial fragmentation; mild atrophy and reduced quality.^69^	Impaired mitophagy; accumulation of damaged mitochondria; severe atrophy.^69^
Implication	Suggests fission overactivity contribute to aging phenotype.	Suggests fission deficit impairs turnover of bad mitochondria.
Examples	Aging ↑ DRP1, more fragmentation.89	*Drp1* knockdown in aged mice → severe dysfunction.^69^

DRP1/*Drp1*: Dynamin-related protein 1.

**Table 3 |:** Summary of mitochondrial mechanisms in muscle homeostasis and aging

Mechanism/process	Function/role	Examples/key players
Mitochondrial biogenesis	Generation of new mitochondria to meet energy demands, involving transcriptional coactivators.	PGC-1α co-activating NRF1/2 and TFAM90; exercise and AMPK signaling.
Proteostasis	Folding and degradation of mitochondrial proteins to prevent dysfunction.	mtUPR: ATF4/5 signaling; chaperones (HSP60, LONP1); proteases (ClpP).^90^
Dynamics (fusion/fission)	Remodeling of mitochondrial networks to segregate damage or optimize function.	Fusion: MFN1/2, OPA1; Fission: DRP1, FIS1. Balance critical in aging.^69^
Mitophagy (autophagy)	Selective degradation of damaged mitochondria via autophagosome-lysosome pathway.	PINK1/PARKIN-mediated pathway; BNIP3/NIX receptors; ULK1 complex.^90^
Calcium homeostasis at MERCs	Regulation of Ca2^+^ transfer between ER/SR and mitochondria, affecting metabolism.	IP3R-VDAC-MCU axis at MAMs; RYR1 leak in aging muscle.^98,145^
Oxidative stress/ROS	Reactive species generation and detoxification affecting mitochondria and ER.	NRF2/KEAP1 pathway; antioxidants; age-related RYR1 oxidation.^145^

AMPK: AMP-activated protein kinase; ATF4: activating transcription factor 4; ER: endoplasmic reticulum; HSP60: heat shock protein 6; IP3R–VDAC–MCU: Inositol 1,4,5 trisphosphate receptor-voltage dependent anion channel-mitochondrial-calcium uniporter; LONP1: Lon protease 1; MAMs: mitochondria-associated membranes; MERCs: mitochondria–endoplasmic reticulum contact sites; MFN1/2: mitofusin 1/2; NRF2/KEAP1: nuclear factor erythroid 2-related factor 2/Kelch-like ECH-associated protein 1; OPA1: optic atrophy 1; ROS: reactive oxygen species; RYR1: ryanodine receptor 1; TFAM: mitochondrial transcription factor A.

**Table 4 |:** Technologies for studying mitochondrial and cellular homeostasis in skeletal muscle

Technology	Application	Advantages/notes
High-resolution microscopy	Visualize mitochondrial morphology (confocal, EM)	Confocal: live imaging of dynamics; EM: ultrastructure. EM revealed MERC alterations in aging.^98^
Fluorescent reporters	Track autophagy/mitophagy (e.g., mitoKeima)	Can quantify mitophagy flux in muscle fibers; requires transgenic models.^146^
Respirometry (Seahorse/Oroboros)	Measure mitochondrial respiration and function	High sensitivity to O_2_ consumption; limited throughput for tissues.^147^
Biochemical assays	Enzyme activities, protein levels (e.g., CS, ATPase)	Standardizable; enzymes like citrate synthase indicates mitochondrial content.^148^
Genetic models	Knockout/overexpression of MQC genes (e.g., DRP1, PGC-1α)	Allow causal tests; species differences may limit translation.^69^
Proteomics/Mass spectrometry	Quantify mitochondrial or MERC protein composition	Unbiased; used to profile muscle MAM proteins in aging.^98^
Live tissue imaging (Ca^2+^ dyes)	Measure Ca^2+^ flux between ER/SR and mitochondria	Reveals functional MERC coupling; limited by dye specificity.^149^

CS: Citrate synthase; DRP1: dynamin-related protein 1; EM: electron microscopy; ER: endoplasmic reticulum; MERC: mitochondria–endoplasmic reticulum contact site; MQC: mitochondrial quality control; PGC-1α: peroxisome proliferator-activated receptor gamma coactivator 1-alpha; SR: sarcoplasmic reticulum.

## Highlights

- Dynamin-related protein 1 (DRP1), a fission protein, interacts with other proteins in a broader interactome, while DRP1 is also affected through post-translational modifications, such as phosphorylation and SUMOylation.
- These downstream and upstream effectors of DRP1 activity are altered in aging muscle and may dictate mitochondrial outcomes in sarcopenia.
- DRP1 may also interact with mitochondria–endoplasmic reticulum contact sites to may play a central role in the progression of sarcopenia.
- There is translational uncertainty about how and when to target DRP1 therapeutically to prevent or reverse sarcopenia.

## Significance

This review provides an overview of the interconnected sarcopenic roles of the fission regulator DRP1, the broader fission protein interactome that interacts with DRP1, mitochondria–endoplasmic reticulum contact sites, and actin cytoskeletal remodeling. Based on recent insights and knowledge within each of these fields, we highlight underexplored pharmacological, lifestyle, and gene therapy strategies aimed at modulating mitochondrial dynamics, highlighting unanswered questions for future interventions.

## Outstanding questions

- Do homotypic (same type) versus heterotypic (different type) fission and fusion events contribute to the formation of altered mitochondrial shapes, and the onset and progression of sarcopenia?
- How do factors including cristae remodeling patterns, cristae orientation and architecture, and spatial orientation of the MICOS complex interconnect with one another and influence mitochondrial function in sarcopenia?
- What roles do proteins responsible for remodeling mitochondrial dynamics (e.g., DRP1, MFN1/2, OPA1) play in affecting mitochondria DNA integrity and alterations, or vice versa? In turn, how do mitochondria DNA mutations, deletions, or copy number changes contribute to mitochondrial dysfunction in sarcopenia?
- What roles, if any, do actin dynamics, such as impaired actin remodeling, play in coordinating fission, fusion, cristae organization, and mitochondrial positioning in sarcopenic muscle fibers?
  What molecular mechanisms, if any, underlie fiber-type–specific (i.e., between type I [oxidative] and type II [glycolytic]) differences in mitochondrial ultrastructure in sarcopenia?
